# Interobserver Agreement on Focused Assessment with Sonography for Trauma in Blunt Abdominal Injury

**DOI:** 10.7759/cureus.2592

**Published:** 2018-05-08

**Authors:** Azeemuddin Muhammad, Adeel A Waheed, Muhammad Ismail Alvi, Noman Khan, Raza Sayani

**Affiliations:** 1 Department of Radiology, The Aga Khan University, Karachi, PAK

**Keywords:** abdominal trauma, trauma sonography, fast, inter-observer agreement, resident

## Abstract

Introduction

Trauma constitutes a major public health problem. Ninty percent of world's fatalities on road occur in low and middle-income countries. Focused assessment with sonography in trauma (FAST) has a key role in trauma investigation, altering subsequent management in a significant number of patients. There is a rising trend of introducing FAST examination to non-radiologists and junior members of healthcare team to hasten triage of trauma patients.

Objective

To determine interobserver agreement on focused assessment with sonography for trauma in blunt abdominal injury between senior and junior residents.

Methods

This cross-sectional study was conducted at Aga Khan University Hospital. Three hundred patients with blunt abdominal trauma meeting inclusion criteria were enrolled. FAST was performed using standard curvilinear 3.5-5 MHz transducer. Agreement between junior and senior residents was measured and Kappa statistic was calculated.

Results

Mean age of the patients included in the study was 30.04 ± 18.09 years. Among these 237 (79%) were male and 63 (21%) were female. Sixteen (5.3%) were positive for intraperitoneal free fluid while the remaining 284 (94.7%) were negative. A total of 19 FAST examinations were reported positive by junior residents, of which 15 (78.9%) were confirmed by a senior resident to be correct, while four (21.05%) were falsely labeled positive by a junior resident. A total of 281 negative FAST examinations were reported by junior residents, of which 280 (99.6%) were confirmed by a senior resident, while only one (0.003%) was falsely labeled negative. Kappa statistic was calculated for inter-observer agreement on FAST examination findings, which showed a kappa value of 0.84 (very good agreement), with a p-value of <0.001.

Conclusion

Our study suggests very good interobserver agreement on FAST examination between senior and junior resident in patients with blunt trauma to the abdomen. Results suggest that FAST can be easily learnt with minimal radiology training and may have greater applicability in trauma.

## Introduction

Road traffic accidents (RTA) are a major health problem causing 1.25 million deaths globally in 2013. According to the Global Status Report on Road Safety by World Health Organization (WHO), 90% of road traffic accident-related fatalities occur in low income and middle-income countries. According to WHO projections, RTA will become the fifth leading cause of death by 2030 [1]. In the first national injury survey in Pakistan, the yearly overall incidence of injury was found to be 41.2 per 1000 persons per year with most injuries affect 16-45 age group [2]. Focused assessment with sonography in trauma (FAST) was originally designed to detect intraperitoneal free fluid although new protocols are being studied. FAST is a rapid bedside technique to detect free fluid suggestive of hemoperitoneum, hemothorax, and hemopericardium. FAST is utilized by radiologists, sonographers, emergency room (ER) physicians and surgeons alike, however, sensitivity for intra-abdominal injury may be lower in less experienced hands [3-5].

FAST is often the first investigation in trauma helping in clinical decision making and changing management in significant number of patients. Currently the widespread use of FAST is limited to radiologists in Pakistan, however, there is a rising trend to introduce FAST to non-radiologists and junior residents to hasten triage of trauma patients [6].

The purpose of this study was to assess interobserver agreement on FAST in blunt abdominal trauma between senior and junior radiology residents.

## Materials and methods

This prospective cross-sectional study was conducted at Aga Khan University Hospital (AKUH) between July 2014 and December 2015. All patients who presented with blunt abdominal trauma to the emergency department of AKUH and underwent FAST examination were included in the study. Patients with penetrating abdominal trauma were excluded from the study.

Three hundred patients with blunt abdominal trauma referred to radiology department were enrolled in the study. FAST was performed in the portable setting in ER or in the departmental setting using the standard curvilinear 3.5-5 MHz transducer by junior radiology resident and reevaluated by senior radiology resident. Outcome variables were measured in terms of the agreement between junior and senior radiology residents. All the information of estimated variable was entered into pre-designed pro forma. Interobserver agreement was calculated using Kappa statistic. Data analysis was performed using SPSS version 21.0 (IBM, Armonk, NY).

## Results

A total of 300 patients were included in this prospective study. Among these, 256 (86%) presented with a history of road traffic accident and 42 (14%) presented with a history of fall. The mean age was 30.04 ± 18.09 years. Among these 237 (79%) were males and 63 (21%) females. The most frequent mode of abdominal injury study was RTA (86%) followed by fall (14%). The most common age group was 16-40 years (35%). FAST findings according to demographic characteristics and mode of injury are shown in Table 1.

**Table 1:** Findings of FAST (Focused assessment with sonography for trauma) examinations. RTA: Road traffic accident.

FAST examination findings			
		Negative	Positive
Gender	Female	61	2
	Male	223	14
Age group	Preschool (<5 yrs)	26	1
	Adolescent (5-15 yrs)	41	1
	Young (16-40 yrs)	102	5
	Middle age (41-60 yrs)	71	3
	Elderly (>61 yrs)	44	6
Mode of Injury	Fall	37	5
	RTA	247	11

Among 300 FAST examinations, 16 (5.3%) were found to be positive for intraperitoneal free fluid while the remaining 284 (94.7%) were negative.

A total 19 FAST examinations were reported positive by junior residents, of which 15 (78.9%) were confirmed to be correct by a senior resident, while four (21.05%) were falsely labeled positive. A total 281 negative FAST examinations were reported by junior residents, of which 280 (99.6%) were confirmed by a senior resident, while only one (0.003%) was falsely labeled negative (Table 2).

**Table 2:** Sensitivity of FAST (Focused assessment with sonography for trauma) examinations.

	Senior resident, n (% age)			
Junior, n (% age)		Positive	Negative	Total
	Positive	15 (5.0%)	4 (1.33%)	19
	Negative	1 (0.33%)	280 (93.33%)	281
	Total	16	284	300

Keeping the findings of the senior resident as a reference standard, the true positive rate for junior residents' FAST examination findings was 80%, true negative rate 99.6%, false positive rate 1.39% and false negative rate was 5.88%.

Kappa statistic was calculated for inter-observer agreement on FAST examination findings, which showed a kappa value of 0.84 (very good agreement), with a p-value of <0.001. The inter-observer agreement on FAST examination findings among year one and senior residents has a kappa value of 0.76 representing good agreement, with a p-value of <0.001. The inter-observer agreement on FAST examination findings among year two and senior residents has a kappa value of 0.94 representing excellent agreement, with a p-value of <0.001.

## Discussion

FAST is a noninvasive bedside investigation to detect free fluid in the peritoneal and pericardial cavity. FAST is a valuable investigation in the initial assessment of blunt abdominal trauma. An extended FAST examination (eFAST) was developed in the mid-2000s for detection of pneumothorax in addition to intraperitoneal free fluid [4].

The presence of free fluid is a highly reliable indicator of the need for laparotomy, however, this is difficult to establish clinically. Findings on physical examination can be misleading in trauma setting as the patient may be unconscious, drowsy or in a state of induced sedation. Diagnostic modalities guiding a clinician in emergency room include FAST, computed tomography (CT) scan and diagnostic peritoneal lavage (DPL). FAST has become the mainstay of trauma assessment due to its reproducibility, non-invasiveness and noninterference with subsequent CT examination. CT and DPL are not favored as the former involves radiation exposure and the latter is an invasive procedure.

FAST can be carried out by radiologists, surgical resident or emergency physician with variable experience and training. FAST has a specificity of 94%–100% for detection of free fluid and has a sensitivity of 72%–93%, however, serial FAST examinations can increase the sensitivity [4].

Jehangir et al. reported a sensitivity of 91% and specificity of 100% of FAST examinations carried out by experienced radiologists in ER [7]. Bhoi et al. reported good agreement with radiologists and high sensitivity of FAST examinations carried out by non-radiologists who attended a three-day workshop on emergency ultrasound [3]. A similar study by McCarter et al. reported 90% accuracy of FAST by trauma surgeons newly trained in the modality [8]. Patelis et al. reported high accuracy of FAST performed by non-radiologists in the detection of hemoperitoneum [9].

False-negative FAST examinations may result from technical errors such as inadequate gain/depth settings, incomplete anatomic interrogation or misinterpretation of anechoic structures as intraperitoneal fluid like urinary bladder, fluid-filled bowel loops or cystic intra-abdominal or adnexal structures. Technical errors and interpretive skills improve with hands-on experience, with improvement in interpretive skills more rapid than image acquisition skills [10].

In our study, we assessed interobserver agreement between senior and junior radiology resident of FAST between junior and senior radiology residents. The junior radiology resident had rotated in ultrasound suite for one month. Results show excellent interobserver agreement (Kappa of 0.84) between junior and senior radiology residents on FAST examination in blunt abdominal trauma. In a similar study, Buzzas et al. reported similar sensitivity and specificity of FAST between surgery and radiology residents [11]. To the best of authors’ knowledge, interobserver agreement on FAST has not been studied previously among different level of radiology residents.

A high true negative rate suggests that FAST can be reliably performed by junior members of trauma team with minimal training. Inclusion of short courses on trauma sonography may be highly useful in rural areas where there is lack of formally trained radiologists. Keeping in view the global trend of rising accidents especially in low-income countries and non-availability of trained radiologists in rural settings, we would suggest short-term trauma ultrasound courses for junior members of trauma response teams such as surgery residents, emergency physicians and nurses.

## Conclusions

There is an excellent interobserver agreement on FAST between senior and junior radiology residents. FAST can be learnt easily without formal radiology training and may have greater applicability in triaging trauma patients.
